# Analysis of Quality Differences in *Rhizoma Musae* From Different Origins Based on Rhizosphere Soil Bacterial Diversity and Metabolomics

**DOI:** 10.1002/fsn3.71958

**Published:** 2026-05-29

**Authors:** Jian Zhang, Xinying Xiong, Jing Lei, Yu Zhang, Yae Yang, Zhongze Wang, Wanyan Shen, Xuegang Luo, Fanzhi Liu, Xiaorong Zhao, Xiaoyu Yang, Peiling Long, Jiandong Liu

**Affiliations:** ^1^ College of Life Sciences and Agri‐Forestry Southwest University of Science and Technology Mianyang China; ^2^ GuiZhou Institute of Subtropical Crops Guizhou Academy of Agricultural Sciences Guiyang China; ^3^ Engineering Research Center of Biomass Materials, Ministry of Education Southwest University of Science and Technology Mianyang China; ^4^ Research and Development Department Guizhou Weikang Zifan Pharmaceutical Co., Ltd. Guiyang China; ^5^ Wangcang County Hospital of Traditional Chinese Medicine Guangyuan China

**Keywords:** metabolomics, quality differences, *Rhizoma Musae*, rhizosphere microorganisms, rhizosphere soil

## Abstract

The origin is a core factor affecting the quality of Chinese medicinal materials. To reveal the intrinsic relationship among soil factors, rhizosphere microorganisms, and quality differences of *Rhizoma Musae* (the dried rhizome of *Musa basjoo*), we systematically explored the formation association and potential underlying mechanisms of *Rhizoma Musae* from different origins using integrated analysis of bacterial diversity and metabolomics. The results showed that the contents of total flavonoids, polysaccharides, total phenols, and alkaloids in *Rhizoma Musae* varied significantly among origins (*p* < 0.05), with samples from GZ showing the highest overall levels. Although no significant difference was observed in rhizosphere bacterial community diversity across origins (*p* > 0.05), the composition of dominant bacterial genera varied distinctly. A total of 571 differential metabolites were identified via metabolomic analysis, which were mainly enriched in phenylpropanoid metabolism, flavonoid biosynthesis, and ABC transporter pathways. Correlation analysis revealed that rhizosphere microorganisms were significantly associated with soil factors transformation and secondary metabolism regulation in *Rhizoma Musae*. In conclusion, soil factors were significantly associated with rhizobacterial community structure at the genus level, and these specific microbial taxa showed significant correlations with the accumulation of plant secondary metabolites related to soil resource utilization; collectively, soil properties and rhizosphere microorganisms showed significant associations with the quality differences of *Rhizoma Musae* from different geographical origins. This study provides a scientific basis for quality evaluation, genuineness association analysis, and putative high‐quality cultivation of *Rhizoma Musae.*

## Graphical abstract

This study integrated bacterial diversity and metabolomics to explore correlations among soil factors, rhizosphere microorganisms, and quality of *Rhizoma Musae* from different origins. Significant differences in active components and 571 differential metabolites were detected, enriched in key secondary metabolism pathways. Soil properties and rhizosphere microbiota are associated with its quality differences, supporting quality evaluation and high‐quality cultivation.

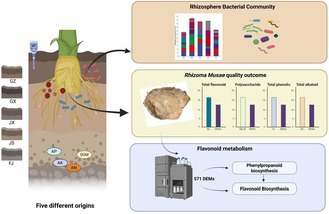

## 1 Introduction

*Musa basjoo* Sied. et Zucc., a perennial herbaceous plant of the *Musaceae* family, is extensively distributed in subtropical regions and cultivated in various provinces of China, including Guangxi, Fujian, Guizhou, and Jiangsu (Editorial Committee of The Flora of China 2004). The medicinal components of *Musa basjoo* primarily consist of its leaves, flowers, and rhizomes. *Rhizoma Musae* (the dried rhizome of *Musa basjoo*), a traditional medicinal and edible material commonly used by the Miao and Buyi ethnic groups in Guizhou Province, China, also serves as an important non‐grain feed resource. At present, this medicinal material has been included in the Quality Standards of Traditional Chinese Medicine and Ethnic Medicine of Guizhou Province (2019 Edition) (Guizhou Provincial Drug Administration 2020). Modern pharmacological investigations have revealed that *Rhizoma Musae* exhibits anti‐inflammatory (Wang et al. 2010), analgesic (Liang et al. 2017), antimicrobial (Wei et al. 2010), and antioxidant effects (Liu et al. 2013). Gukang capsules, which are primarily derived from *Rhizoma Musae*, have demonstrated significant therapeutic efficacy in the treatment of fractures, osteoarthritis, and other related conditions (Zhu et al. 2022).

Soil is regarded as one of the primary factors influencing the quality of traditional Chinese medicinal materials. Specific soil conditions were significantly associated with the synthesis and storage of secondary metabolites in medicinal plants, which may be related to their distinct pharmacological properties (W. Liu et al. 2020). For example, the availability of phosphorus (AP) in the soil has been shown to affect both biomass and artemisinin production in *Artemisia annua* L. (Wan et al. 2024). The pH of the soil is a significant factor influencing the growth and material accumulation in *Angelica sinensis* (F. Xu et al. 2020). Available iron in the soil has been found to promote the accumulation of flavonoid compounds in *Radix Astragali* (Bi et al. 2020). Microbial communities, as integral components of soil, serve a pivotal function in nutrient cycling, fertility formation, and the enhancement of the ecological environment (Zou et al. 2022), while also showing significant associations with plant growth and development (Fierer et al. 2012). The rhizosphere, which represents the most active zone for material and energy exchange between plant roots and soil, serves as a critical interface in plant–soil interactions (Lv et al. 2023). Within this rhizosphere zone, root exudates have the ability to influence the composition and growth of soil bacteria. In exchange for nutrients, soil bacteria enhance the bioavailability of nutrients for plants (Henneron et al. 2020).

The primary and secondary metabolites of medicinal plants exhibit considerable complexity and diversity, which complicates the analysis of their biosynthetic pathways and regulatory associations using traditional analytical approaches (Nyakudya et al. 2020). Multi‐omics integrated analysis has emerged as a fundamental research tool for exploring the medicinal components of traditional Chinese medicines (Zhou et al. 2024). Microbiomics employs high‐throughput sequencing technologies to investigate the composition, function, and variation patterns of microbial communities in specific environments (Ahsan et al. 2024). Metabolomics facilitates the processing and identification of metabolites in biological samples through high‐throughput detection and quantitative analysis techniques (Tuyiringire et al. 2018). Research on *Rhizoma Musae* remains limited, with existing studies primarily concentrating on the identification, isolation, and purification of individual active components (X. Xu et al. 2020; Xu et al. 2015). Furthermore, no comparative studies have been reported regarding the quality of *Rhizoma Musae* from different origins. To date, the knowledge gap concerning comparative quality assessment across different geographical origins remains unaddressed. Current work has also not adequately distinguished observed quality variation, its potential associations with soil properties and rhizosphere microbial communities, and the underlying causal mechanisms, which have not been experimentally demonstrated. This study employed an integrated analysis combining microbiomics and metabolomics to investigate the differential components and underlying associations of *Rhizoma Musae* from distinct origins. An association network linking soil factors, rhizosphere soil bacteria, and metabolites of *Rhizoma Musae* was constructed, providing a basis for the quality identification and optimized cultivation techniques of this medicinal material.

## 2 Materials and Methods

### 2.1 Experimental Materials and Sample Preparation

Samples of wild *Rhizoma Musae* with a pseudostem diameter of approximately 6 cm were collected from one field site per province (five major production areas, Table 1): Guizhou Province, Guangxi Province, Jiangxi Province, Jiangsu Province, and Fujian Province. All samples were identified as *Rhizoma Musae* according to the Quality Standards of Traditional Chinese Medicine and Ethnic Medicine of Guizhou Province (Guizhou Provincial Drug Administration 2020). Post‐harvest handling and processing were completely consistent across all sites. The corms were sectioned into 5 mm‐thick slices, dried to a constant weight, frozen in liquid nitrogen, and stored at −80°C for subsequent analysis.

**TABLE 1:** Geographic location information of *Rhizoma Musae* samples collected from five provinces in China.

No.	Sampling location	Altitude (m)	N	E
GZ (1–5)	Qiandongnan Prefecture, Guizhou Province	551	26°87′63″	109°21′28″
GX (1–5)	Qinzhou City, Guangxi Zhuang Autonomous Region	65	22°31′25″	109°29′22″
JX (1–5)	Yichun City, Jiangxi Province	276	28°53′36″	114°90′60″
JS (1–5)	Suqian City, Jiangsu Province	66	33°89′21″	118°54′69″
FJ (1–5)	Nanping City, Fujian Province	1067	27°45′26″	117°26′87″

### 2.2 Soil Property Analysis

Following the procedure outlined by Ni et al. (2008), soil samples were collected from various locations and preserved at 4°C for subsequent physicochemical analysis. The pH of the soil was determined using a pH meter (LC‐MP‐31, LiChen, China). The soil organic matter (SOM) content was assessed based on the method described by Yuan, calculated by multiplying the organic carbon content by 1.724 (Yuan et al. 2024), with the unit expressed as mg/g. Ammonium nitrogen (AN) was quantified using the indophenol blue colorimetric method (Liu et al. 2022), with a unit of mg/g. AP and available potassium (AK) were measured according to the methods of Bao (1999), with units of μmol/g for AP and mg/kg for AK, respectively. Three technical replicates were performed for each soil sample to ensure analytical precision.

### 2.3 Determination of Major Medicinal Components

To explore the variations in medicinal constituents of *Rhizoma Musae* originating from diverse sources, commercial test kits provided by Jiangsu Aidisheng Biotechnology Co. Ltd. (Yancheng, China) were employed. The kits, identified as ADS‐W‐KY007‐96, ADS‐W‐TDX061, ADS‐W‐KY008‐96, and ADS‐W‐QT016, were used for the determination of total flavonoids, polysaccharides, total phenols, and alkaloids, respectively. Detection wavelengths were established at 510, 488, 760, and 415 nm. Five biological replicates were conducted for each sample source.

### 2.4 Analysis of Bacterial Communities in Rhizosphere Soil

Following the method of X. Liu et al. (2020), rhizosphere soil samples were obtained through gentle washing of the root system with water, followed by centrifugation, and subsequently stored at −80°C. Total genomic DNA was extracted using the OMEGA Soil DNA Kit (M5635‐02, OMEGA Bio‐Tek, Norcross, GA, USA) and preserved at −20°C. The concentration of DNA was quantified by agarose gel electrophoresis and with the NanoDrop2000 (Thermo Fisher Scientific, Waltham, MA, USA). For PCR amplification, bacterial 16S rRNA V3‐V4 region‐specific primers, 338F (5′‐ACTCCTACGGGAGGCAGCA‐3′) and 806R (5′‐GGACTACHVGGGTWTCTAAT‐3′) were employed. Sequencing was carried out on the Illumina NovaSeq 6000 platform at Personal Bio (Nanjing, China). Three biological replicates were used per origin for rhizosphere bacterial community analysis.

The raw sequences were processed using the QIIME2 analysis platform (https://qiime2.org). Decoding was performed using the demux plugin, while primer removal was conducted with the cutadapt plugin. Following this, the DADA2 plugin was employed for quality filtering, denoising, merging, and chimera removal. The sequences were then processed to generate amplicon sequence variants (ASVs) and abundance tables. Representative ASV sequences were selected and annotated by alignment with the Silva database to assign taxonomic information. Alpha diversity was assessed using the Chao1 index, Good's coverage index, Simpson index, and Shannon index. Beta diversity was evaluated via principal coordinate analysis (PCoA) and nonmetric multidimensional scaling (NMDS) analysis. To identify markedly different species across groups, linear discriminant analysis effect size (LEfSe) was conducted.

### 2.5 Metabolomic Analysis of Dried *Rhizoma Musae*

To examine metabolite variations in *Rhizoma Musae* from different origins, metabolomic analysis was conducted on samples collected from five locations, each with three biological replicates. The analysis was carried out by Personal Bio (Nanjing, China). Metabolite structural identification was performed using a combination of a local self‐built biological database and public databases including HMDB, Metlin, MassBank, and mzCloud. Annotation was achieved by matching retention time, molecular mass (mass error < 10 ppm), MS/MS fragmentation spectra, and collision energy. All identification results were strictly verified and confirmed by manual secondary inspection. In the present study, all metabolites were identified at Level 2 in accordance with the Metabolomics Standards Initiative.

Original mass spectrometry data (wiff.scan files) were converted into MzXML format for subsequent processing. Principal component analysis (PCA) and partial least squares discriminant analysis (PLS‐DA) were executed using the R package Ropls (Thévenot et al. 2015) for dimensionality reduction. Metabolite abundance clustering was performed with the R package Pheatmap (V1.0.12). Kyoto Encyclopedia of Genes and Genomes (KEGG) enrichment analysis of differential metabolites was conducted using clusterProfiler (V4.6.0) to identify markedly enriched metabolic pathways. Spearman correlation coefficients were used to evaluate correlations.

### 2.6 Statistical Analysis

Statistical analysis was performed using SPSS 27.0 (New York, USA). Data were first tested for normality (Shapiro–Wilk test) and homogeneity of variance (Levene's test). One‐way analysis of variance (ANOVA) was used for multi‐group comparisons, followed by LSD post hoc test. The Benjamini‐Hochberg (FDR) method was applied for multiple testing correction. All data were expressed as mean ± standard deviation, and *p* < 0.05 was considered statistically significant.

## 3 Results

### 3.1 Determination Results of Soil Factors at Different Sampling Sites

The soil factor measurements procured from various sampling sites of *Rhizoma Musae* are summarized in Table 2. Soil pH values ranged between 5.81 and 5.94, indicating slightly acidic conditions. Among the sampled regions, the JS group exhibited the lowest pH, which was markedly lower than those of the other four locations (*p* < 0.05). The soil in the JS group exhibited the highest concentrations of AP, AK, and SOM, at 24.88 μmol/g, 535.65 mg/kg, and 35.93 mg/g, respectively, with highly significant differences compared to the other sites. The JX group contained the highest AN content, at 15.01 mg/g, markedly surpassing the other four locations (*p* < 0.05).

**TABLE 2:** Soil factor measurement results of *Rhizoma Musae* samples from five production areas.

Soil factor	GZ	GX	JX	JS	FJ
pH	5.81 ± 0.14^a^	5.94 ± 0.28^a^	5.70 ± 0.08^ab^	5.43 ± 0.16^b^	5.92 ± 0.2^a^
AP (μmol/g)	0.40 ± 0.07^b^	1.13 ± 0.28^b^	1.15 ± 0.55^b^	24.88 ± 3.72^a^	2.27 ± 2.7^b^
AK (mg/kg)	42.88 ± 1.11^c^	309.33 ± 166.94^b^	108.29 ± 60.8^bc^	535.65 ± 44.9^a^	170.33 ± 175.65^bc^
SOM (mg/g)	17.30 ± 2.12^b^	18.92 ± 10.35^b^	22.78 ± 10.19^b^	35.93 ± 4.4^a^	16.06 ± 2.78^b^
AN (mg/g)	9.07 ± 2.04^bc^	8.52 ± 2.45^bc^	15.01 ± 2.9^a^	10.17 ± 2.65^b^	5.70 ± 0.37^c^

*Note:* Different letters in the same line indicate significant differences, *p* < 0.05 (*n* = 3).

Abbreviations: AK, available potassium; AN, ammonium nitrogen; AP, available phosphorus; pH, soil pH; SOM, soil organic matter.

### 3.2 Results of Medicinal Component Analysis

**FIGURE 1:**
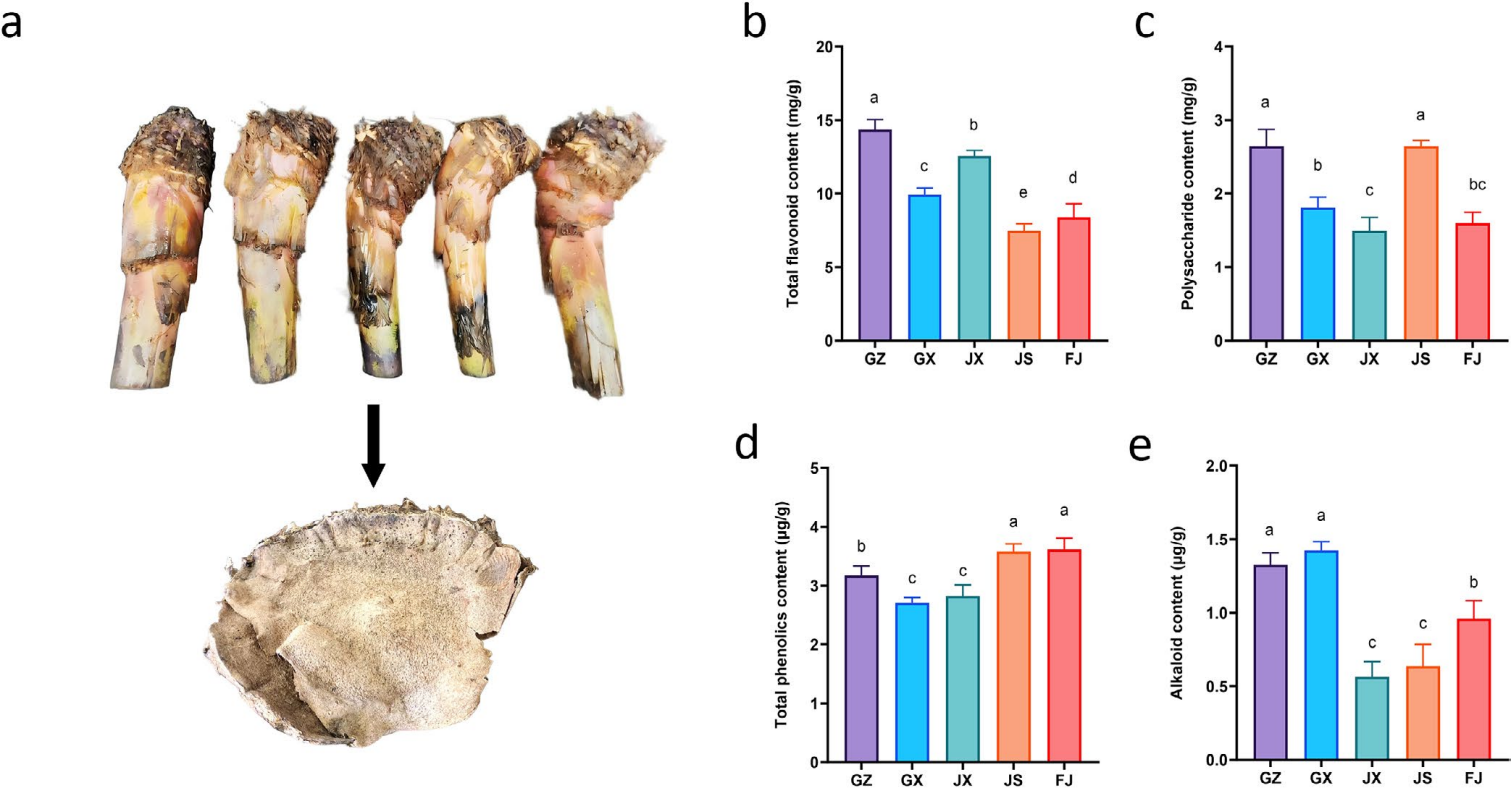
Comparison of medicinal components in *Rhizoma Musae* from different origins. Morphology of fresh *Rhizoma Musae* and its dried slices (a); (b) Total flavonoid content; (c) Polysaccharide content; (d) Total phenolic content; (e) Total alkaloid content. Different lowercase letters above the bars indicate statistically significant differences (*p* < 0.05) among groups, while bars sharing the same letter show no significant difference, based on one‐way ANOVA followed by Duncan’s multiple range test.

Figure 1 shows the determination results of major bioactive components in dried *Rhizoma Musae* from different producing areas. The morphologies of fresh and dried *Rhizoma Musae* were shown in Figure 1a,b, respectively. The total flavonoid content (Figure 1c) in the GZ group was the highest, recorded at 14.37 ± 0.37 mg/g, markedly surpassing that of the JS group by 92.21% (*p* < 0.05). In terms of polysaccharide content (Figure 1d), the GZ and JS groups exhibited notably higher levels, with values of 2.64 ± 0.23 and 2.64 ± 0.08 mg/g, respectively, in comparison to the remaining three origins (*p* < 0.05). The lowest polysaccharide content was found in the JX group, at 1.50 ± 0.18 mg/g. For total phenolic content (Figure 1e), the FJ and JX groups displayed markedly higher levels, with values of 3.61 ± 0.19 and 3.58 ± 0.13 μg/g, respectively, compared to the other origins (*p* < 0.05). Regarding alkaloid content (Figure 1f), samples from the GX and GZ origins contained markedly higher levels, at 1.42 ± 0.59 and 1.33 ± 0.08 μg/g, respectively, compared to the other three origins, while the JX origin showed the lowest alkaloid content at 0.56 ± 0.10 μg/g (*p* < 0.05).

### 3.3 Analysis of Bacterial Diversity in Rhizosphere Soil

**FIGURE 2:**
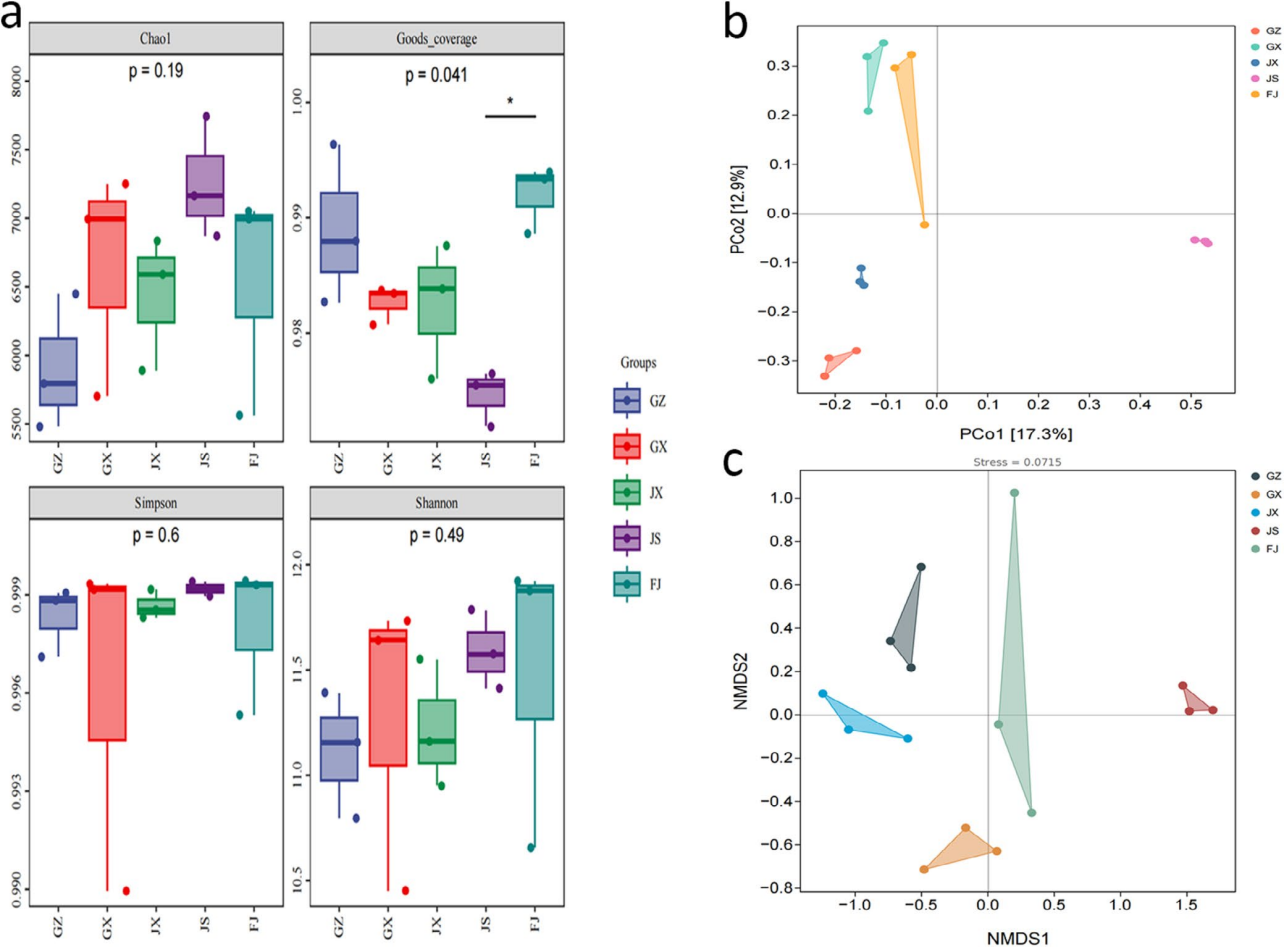
Analysis of bacterial communities and diversity in rhizosphere soil of *Rhizoma Musae*. (a) Alpha diversity indices. (b) PCoA analysis (Bray–Curtis distance, *n* = 3). (c) NMDS analysis (Bray–Curtis distance, *n* = 3). * in panel (a) indicates a statistically significant difference (*p* < 0.05) between groups, as determined by the corresponding statistical test.

The bacterial communities in the rhizosphere soil of *Rhizoma Musae* from various regions were analyzed using 16S rRNA sequencing. In the assessment of alpha diversity, the Chao1 index, Good's coverage index, Simpson index, and Shannon index were employed to characterize the richness, coverage, and diversity of the bacterial communities. As illustrated in Figure 2a, no significant differences were detected in the abundance and diversity of rhizosphere soil microorganisms (the Chao1 index, Simpson index, and Shannon index) across the five regions (*p* > 0.05). The Good's coverage indices exceeded 97% in all cases, suggesting adequate sequencing depth and a reliable representation of the sample conditions. To assess microbial variations between the samples, beta diversity analysis was performed. The PCoA based on Bray–Curtis dissimilarity (Figure 2b) demonstrated clear differentiation among the five groups, with the GZ and JS groups showing the most pronounced separation, while the GX and FJ groups displayed the highest level of similarity. Furthermore, the results from the NMDS analysis (Figure 2c) corroborated the PCoA outcomes. PERMANOVA analysis (999 permutations) confirmed a highly significant overall difference in rhizosphere bacterial community structure among the five origins (pseudo‐*F* = 2.317, *p* = 0.001, Table S1).

**FIGURE 3:**
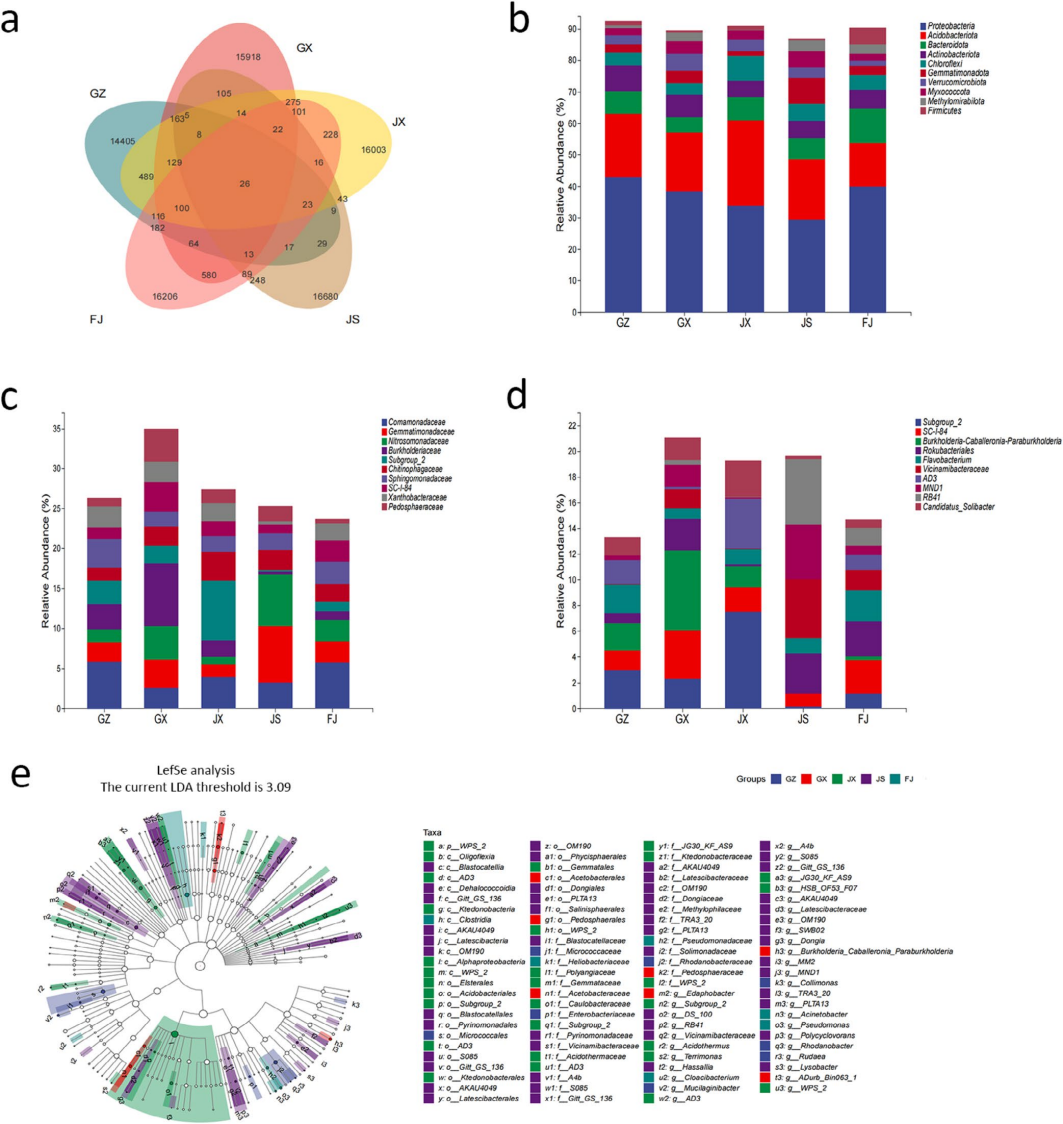
Differential analysis of rhizosphere soil bacteria in *Rhizoma Musae*. (a) Venn diagram based on ASVs. Relative abundance of rhizosphere soil bacteria at phylum (b), family (c), and genus (d) levels. (e) LEfSe analysis showing significantly different microbial taxa among groups (LDA > 3.09, *n* = 3).

Non‐redundant ASV abundances were generated after DADA2 denoising. The distribution of bacterial ASV abundances within the rhizosphere soil of *Rhizoma Musae* across various regions is illustrated in Figure 3a. A total of 26 shared bacterial ASV were identified among the regions. At the phylum level, the bacterial composition in the rhizosphere soil was predominantly represented by *Proteobacteria*, *Acidobacteriota*, and *Bacteroidota* (Figure 3b). At the family level (Figure 3c), the *Comamonadaceae* family displayed the highest relative abundance in the GZ and FJ groups, with values of 5.81% and 5.78%, respectively. In contrast, the JS group exhibited the highest relative abundances of *Gemmatimonadaceae* and *Nitrosomonadaceae*, at 7.04% and 6.48%, respectively. The JX group was characterized by the highest relative abundance of unclassified *Acidobacteriae* (Subgroup_2), reaching 7.55%. The GX group had the highest relative abundance of *Burkholderiaceae* at 7.78%. At the genus level (Figure 3d), the most abundant bacterial genera in the rhizosphere soil of *Rhizoma Musae* included *unclassified Acidobacteriae* (*Subgroup_2*), *SC‐I‐84*, *Burkholderia‐Caballeronia‐Paraburkholderia*, *Rokubacteriales*, *Flavobacterium*, *Vicinamibacteraceae*, *AD3*, *MND1*, *RB41*, and *Candidatus_Solibacter*. The relative abundances of bacterial genera varied across the groups. The GZ and JX groups were predominantly comprised of *unclassified Acidobacteriae* (*Subgroup_2*), with maximum relative abundances of 2.98% and 7.55%, respectively. The GX group displayed the highest relative abundance of *Burkholderia‐Caballeronia‐Paraburkholderia* at 6.19%, while the JS group showed the highest relative abundance of *RB41* at 5.12%. The FJ group had the highest relative abundance of *Rokubacteriales* at 2.73%.

To characterize the microbial community profiles across different regions, linear discriminant analysis (LDA) in combination with LEfSe analysis was employed, resulting in the identification of 99 bacterial genera with significant differences (LDA > 3.09, *p* < 0.05) (Figure 3e). Among these, the GZ group was found to have 10 biomarkers, such as *Rhodanobacter*, *Micrococcales*, and *Enterobacteriaceae*. The GX group exhibited 7 biomarkers, including *Acetobacteraceae*, *Pedosphaeraceae*, and *Edaphobacter*. The JX group was characterized by 30 biomarkers, including *Alphaproteobacteria* and *Acidobacteriales*. The JS group displayed 48 biomarkers, such as RB41, *Vicinamibacteraceae*, and *Lysobacter*. The FJ group was found to contain 5 biomarkers, including *Cloacibacterium*, *Acinetobacter*, and *Pseudomonas*.

### 3.4 Metabolomic Comparison of *Rhizoma Musae* From Different Origins

**FIGURE 4:**
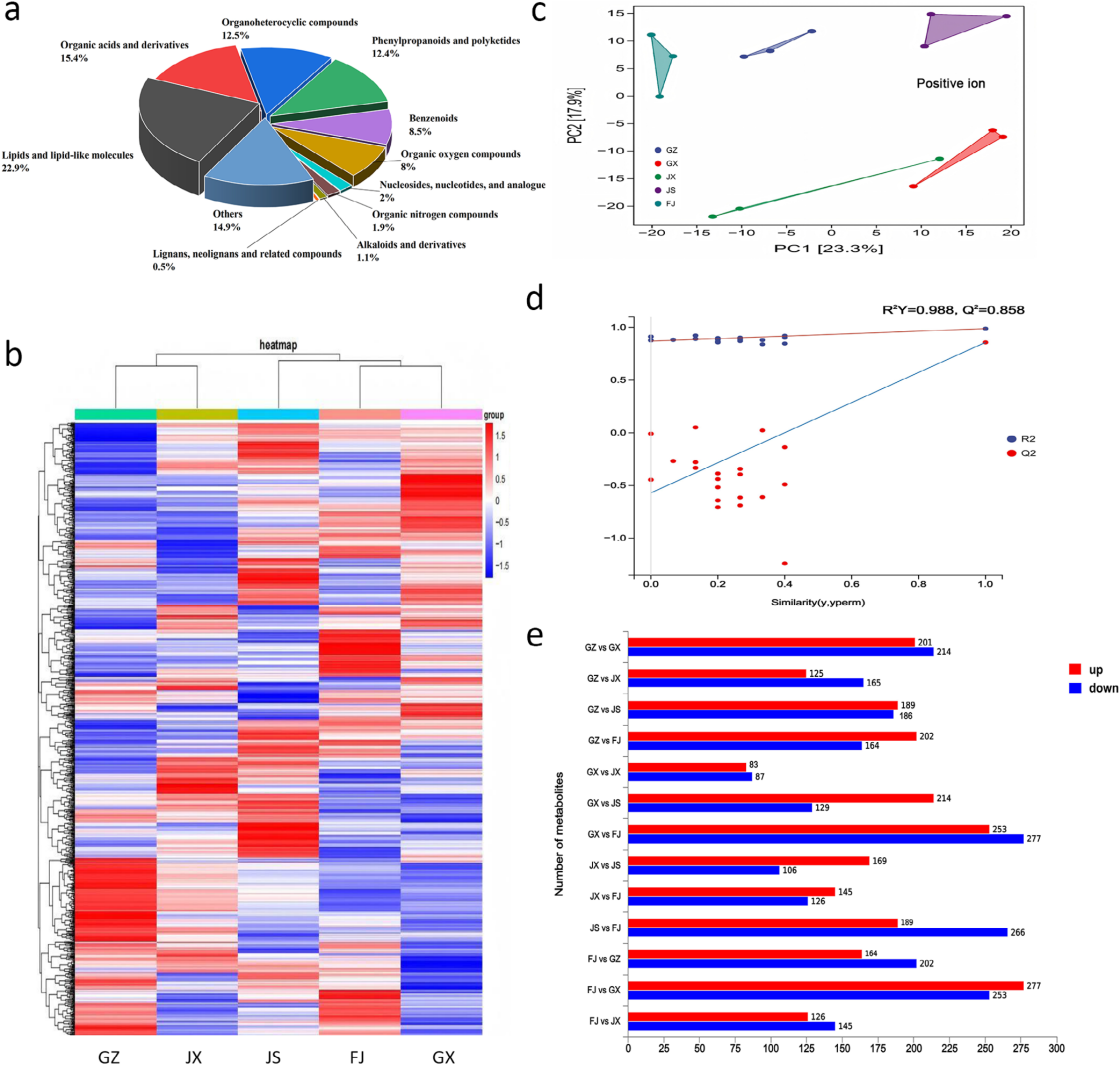
Metabolomic analysis of *Rhizoma Musae* from different origins. (a) Classification of metabolites. (b) Metabolite heatmap (Euclidean distance, *Z*‐score normalized). (c) PCA analysis in positive modes (*Z*‐score normalized, *n* = 3). (d) PLS‐DA score plot of metabolic profiles of *Rhizoma Musae* from five origins. (e) Comparison of up‐regulated and down‐regulated differentially expressed metabolites in *Rhizoma Musae* from different origins (VIP > 1, *p* < 0.05).

Non‐targeted metabolomics analysis based on liquid chromatography‐mass spectrometry was conducted to explore the metabolic variations in *Rhizoma Musae* originating from different regions, leading to the identification of 1822 metabolites (Table S2), which were categorized into 11 groups according to their established structures (Figure 4a). Among these metabolites, 417 were classified as lipids and lipid‐like molecules, 280 as organic acids and their derivatives, 227 as organoheterocyclic compounds, 226 as phenylpropanoids and polyketides, 154 as benzenoids, 145 as organic oxygen compounds, 36 as nucleosides, nucleotides, and analogues, 34 as organic nitrogen compounds, 20 as alkaloids and their derivatives, 10 as lignans, neolignans, and related compounds, and 273 as others.

Based on the Euclidean distance algorithm, the heatmap analysis of metabolite clustering, revealed notable variations in the metabolic profiles across samples from different origins (Figure 4b). The PCA highlighted distinguishable differences among metabolites from the five origins, forming five distinct clusters (Figure 4c). In the positive ion mode, the first and second principal components accounted for 23.3% and 17.9% of the total variance. The biological replicates from each origin exhibited tight clustering, indicating high reproducibility and similarity within each group. The PLS‐DA model (Figure 4d) showed excellent performance in discriminating samples from five origins, with *R* ^2^ *Y* = 0.988, and *Q* ^2^ = 0.858, indicating no overfitting. Based on the combined criteria of *p* < 0.05, VIP > 1, and FDR < 0.05, 571 differentially expressed metabolites (DEMs) were ultimately identified (Table S3). The regulation patterns of these DEMs are illustrated in Figure 4e. Four comparison groups were established using the GX group as a reference, and the top five DEMs were selected based on the descending order of *p* < 0.05 and VIP values, as depicted in Table 3. Among these DEMs, 4‐hydroxy‐3‐(4‐hydroxyphenoxy)benzoic acid exhibited increased abundance in all four comparison groups compared to the GX group. Additionally, (1r,13s,15s,18s)‐5,7‐dioxa‐12‐azapentacyclo[10.5.2.0^1^,^13^.0^2^,^10^.0^4^,^8^]nonadeca‐2,4(8),9,16‐tetraene‐11,15,18‐triol was up‐regulated in both the GZ vs. GX and JS vs. GX comparisons. Similarly, 7‐[(2S,3R,4S,5S,6R)‐4,5‐dihydroxy‐6‐(hydroxymethyl)‐3‐[(2S,3R,4R,5R,6S)‐3,4,5‐trihydroxy‐6‐methyloxan‐2‐yl]oxyoxan‐2‐yl]oxy‐5‐hydroxy‐2‐(4‐hydroxyphenyl)‐3‐[(2S,3R,4S,5S,6R)‐3,4,5‐trihydroxy‐6‐[[(2S,3R,4R,5R,6S)‐3,4,5‐trihydroxy‐6‐methyloxan‐2‐yl]oxymethyl]oxan‐2‐yl]oxychromen‐4‐one was found to be up‐regulated in both the GZ vs. GX and FJ vs. GX groups.

**TABLE 3:** Information of the top three differential metabolites ranked by VIP values in four comparison groups.

Comparison groups	DEMs	Compounds	log_2_ (fold change)	*p*	VIP	Classification	Regulation
GZ vs. GX	415	4‐hydroxy‐3‐(4‐hydroxyphenoxy)benzoic acid	4.55	3.67E−04	1.61	Benzenoids	Up
		(1r,13s,15s,18s)‐5,7‐dioxa‐12‐azapentacyclo[10.5.2.0^1^,^13^.0^2^,^10^.0^4^,^8^]nonadeca‐2,4(8),9,16‐tetraene‐11,15,18‐triol	7.21	3.73E−04	1.61	Alkaloids and derivatives	Up
		7‐[(2S,3R,4S,5S,6R)‐4,5‐dihydroxy‐6‐(hydroxymethyl)‐3‐[(2S,3R,4R,5R,6S)‐3,4,5‐trihydroxy‐6‐methyloxan‐2‐yl]oxyoxan‐2‐yl]oxy‐5‐hydroxy‐2‐(4‐hydroxyphenyl)‐3‐[(2S,3R,4S,5S,6R)‐3,4,5‐trihydroxy‐6‐[[(2S,3R,4R,5R,6S)‐3,4,5‐trihydroxy‐6‐methyloxan‐2‐yl]oxymethyl]oxan‐2‐yl]oxychromen‐4‐one	−5.02	2.69E−04	1.61	Phenylpropanoids and polyketides	Down
JX vs. GX	171	4‐Hydrazino‐5‐phenylthieno[2,3‐d]pyrimidine	−4.49	6.12E−04	1.73	Organoheterocyclic compounds	Down
		3‐oxo‐tetradecanoic acid	−4.71	2.01E−03	1.70	Lipids and lipid‐like molecules	Down
		4‐hydroxy‐3‐(4‐hydroxyphenoxy)benzoic acid	3.80	2.15E−04	1.70	Benzenoids	Up
JS vs. GX	343	(1r,13s,15s,18s)‐5,7‐dioxa‐12‐azapentacyclo[10.5.2.0^1^,^13^.0^2^,^10^.0^4^,^8^]nonadeca‐2,4(8),9,16‐tetraene‐11,15,18‐triol	6.73	2.07E−05	1.64	Alkaloids and derivatives	Up
		Thr‐Ile	−1.25	7.43E−04	1.64	Organic acids and derivatives	Down
		2‐[(4,5‐dihydroxy‐6‐{[6‐hydroxy‐7,9,13‐trimethyl‐6‐(3‐methyl‐4‐{[3,4,5‐trihydroxy‐6‐(hydroxymethyl)oxan‐2‐yl]oxy}butyl)‐5‐oxapentacyclo[10.8.0.0^2^,^9^.0^4^,^8^.0^13^,^18^]icos‐18‐en‐16‐yl]oxy}‐2‐(hydroxymethyl)oxan‐3‐yl)oxy]‐6‐(hydroxymethyl)oxane‐3,4,5‐triol	−7.43	5.43E−04	1.64	Lipids and lipid‐like molecules	Up
FJ vs. GX	530	7‐[(2S,3R,4S,5S,6R)‐4,5‐dihydroxy‐6‐(hydroxymethyl)‐3‐[(2S,3R,4R,5R,6S)‐3,4,5‐trihydroxy‐6‐methyloxan‐2‐yl]oxyoxan‐2‐yl]oxy‐5‐hydroxy‐2‐(4‐hydroxyphenyl)‐3‐[(2S,3R,4S,5S,6R)‐3,4,5‐trihydroxy‐6‐[[(2S,3R,4R,5R,6S)‐3,4,5‐trihydroxy‐6‐methyloxan‐2‐yl]oxymethyl]oxan‐2‐yl]oxychromen‐4‐one	−6.10	1.69E−06	1.48	Phenylpropanoids and polyketides	Down
		methyl 3‐{[(2s,3r,4s,5s,6r)‐3,4,5‐trihydroxy‐6‐(hydroxymethyl)oxan‐2‐yl]oxy}benzoate	4.50	1.04E−03	1.48	Organic oxygen compounds	Up
		Eriodictyol	2.86	8.83E−05	1.48	Phenylpropanoids and polyketides	Up

**FIGURE 5:**
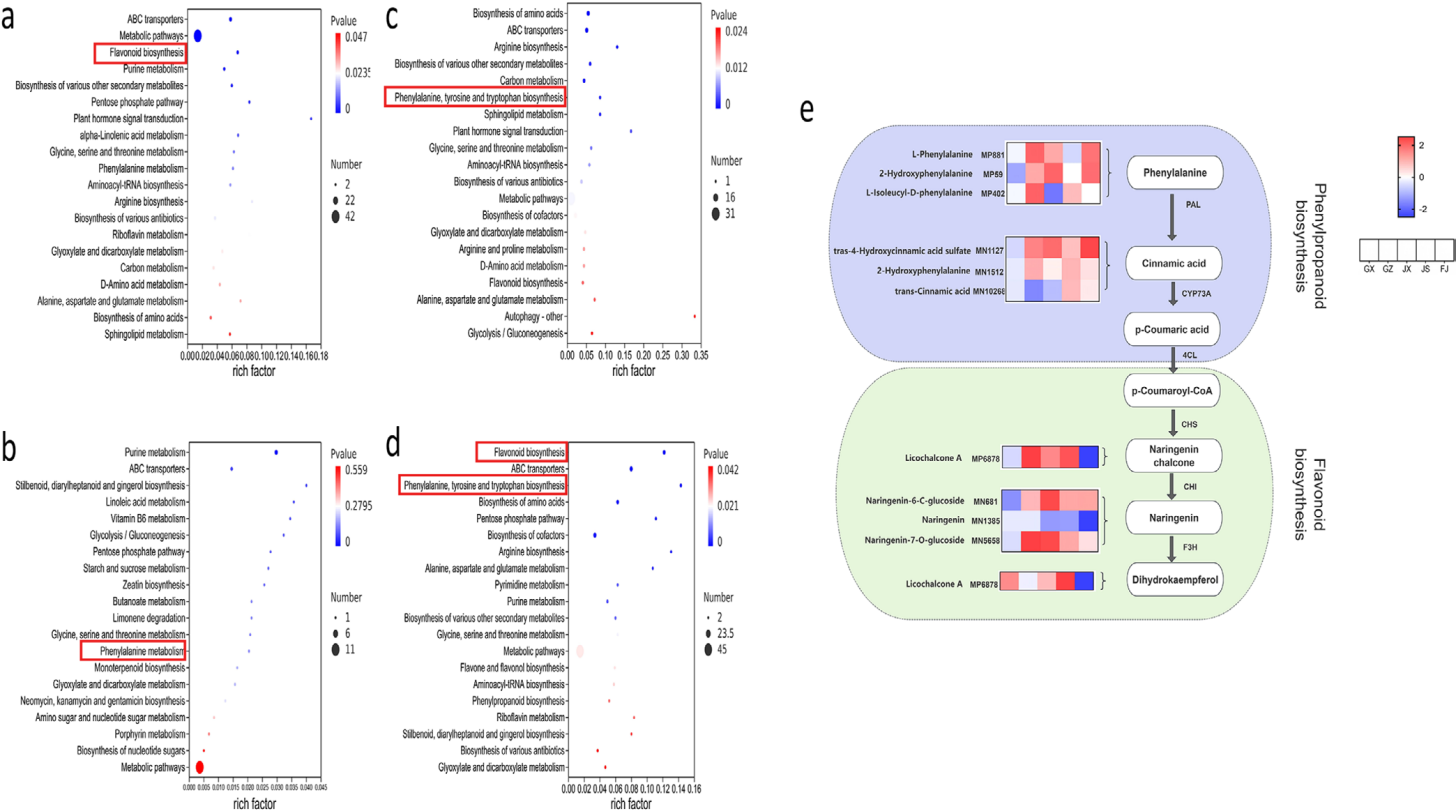
KEGG pathway enrichment analysis of DEMs in *Rhizoma Musae* between pairwise comparison groups. (a) GZ vs. GX; (b) JX vs. GX; (c) JS vs. GX; (d) FJ vs. GX. (e) Phenylpropanoid and flavonoid biosynthesis.

KEGG enrichment analysis was conducted on the top 20 DEMs across the four comparison groups, as these metabolites contributed most to group separation and represented the key metabolic differences among origins (Figure 5). In GZ vs. GX group (Figure 5a), DEMs were notably enriched in pathways such as flavonoid biosynthesis, purine metabolism and Biosynthesis of various other secondary metabolites. For the JX vs. GX comparison group (Figure 5b), significant enrichment was observed in pathways associated with purine metabolism, stilbenoid, diarylheptanoid and gingerol biosynthesis and linoleicacid metabolism. In the JS vs. GX comparison group (Figure 5c), DEMs exhibited marked enrichment in pathways related to biosynthesis of amino acids, arginine biosynthesis and biosynthesisof various othersecondarymetabolites. The most pronounced differences in pathway enrichment were noted in the FJ vs. GX comparison group (Figure 5d), where DEMs were markedly enriched in flavonoid biosynthesis, phenylalanine, tyrosine, and biosynthesis of amino acids. Common enrichment of DEMs from all four comparison groups was observed in pathways related to phenylalanine metabolism, flavonoid biosynthesis, ABC transporters, glycine, serine, and threonine metabolism. Compared with the GX group, analysis of the relative abundances of key metabolites in the phenylpropanoid and flavonoid biosynthesis pathways revealed significant origin‐specific differences in the relative abundances from different producing areas (Figure 5e). L‐phenylalanine derivatives (e.g., MP831, MP589) and cinnamic acid derivatives (e.g., MN1127, MN10268) in the phenylpropanoid metabolism stage; chalcone (Licochalcone A, MP6878), flavanone (Naringenin, MN1385), and their glycoside derivatives (Naringenin‐6‐C‐glucoside, MN681; Naringenin‐7‐O‐glucoside, MN6568) in the flavonoid metabolism stage. These findings unravel shared metabolic adaptation patterns of *Rhizoma Musae* from different habitats and clarify their flavonoid metabolic differences, providing clear pathway targets for subsequent elucidation of quality differences among origins. These results demonstrate significant differences in the metabolic regulation of the phenylpropanoid‐flavonoid biosynthesis pathway among *Rhizoma Musae* from different producing areas, providing a metabolic basis for further elucidating the impact of producing areas on medicinal material quality.

### 3.5 Association Analysis of Soil Factors, Rhizosphere Soil Bacterial Communities, and Metabolites in *Rhizoma Musae*

**FIGURE 6:**
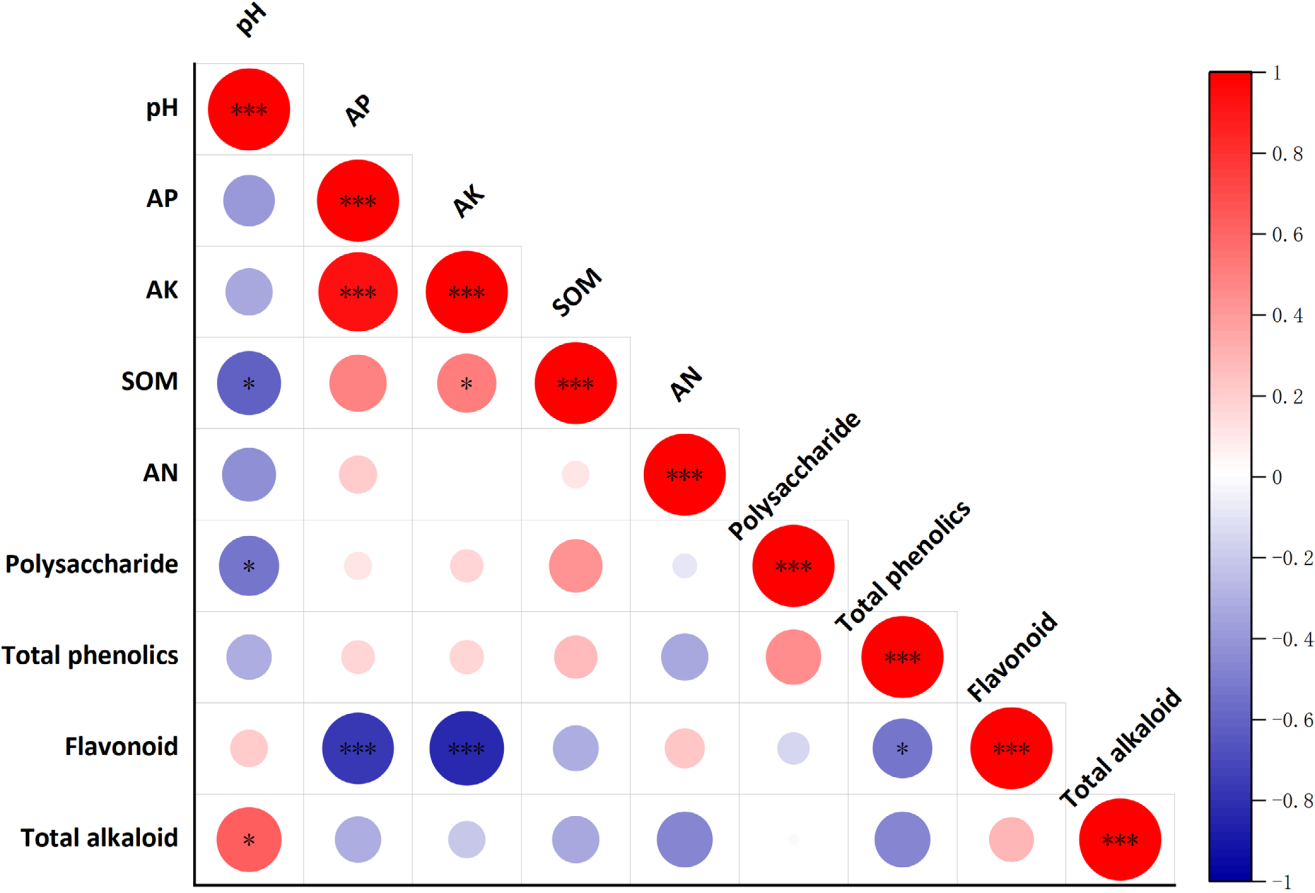
Heatmap analysis of correlations between soil factors and medicinal components of *Rhizoma Musae* from different regions. *p ≤ 0.05; ***p ≤ 0.001.

To explore the relationships between soil factors from various origins and the medicinal constituents of *Rhizoma Musae*, Spearman correlation analysis was performed, as illustrated in Figure 6. A significant positive correlation was observed between pH and total alkaloid content (*p* < 0.05), whereas a significant negative correlation with polysaccharide content was noted (*p* < 0.05). Both AP and AK concentrations were found to have highly significant negative correlations with flavonoid content (*p* < 0.001).

**FIGURE 7:**
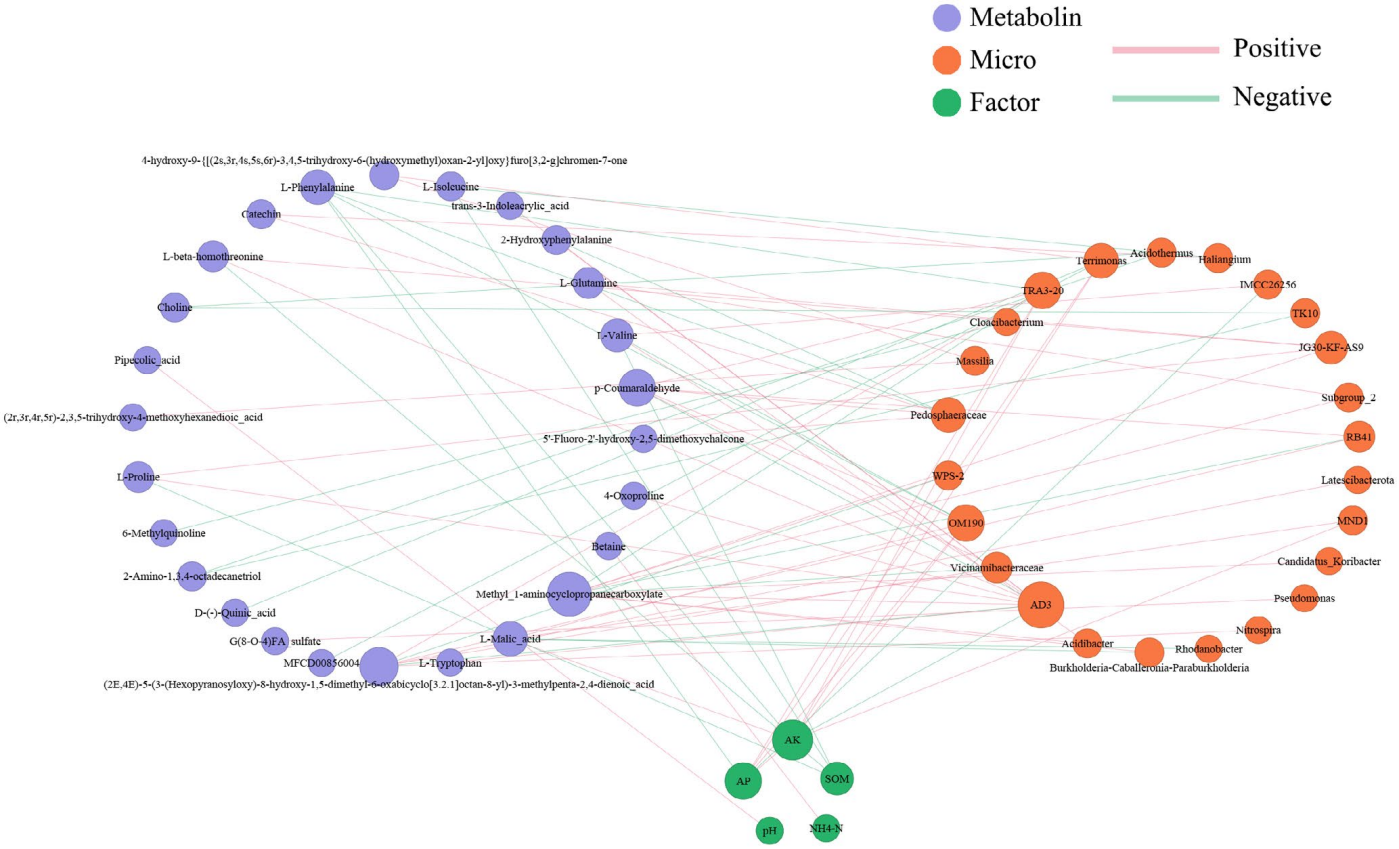
Three‐layer association network among soil factors, top 50 differential bacterial genera, and top 50 DEMs (Spearman |*rho*| > 0.7, *p* < 0.01).

To further elucidate the interrelations between soil factors, rhizosphere bacteria, and metabolites in *Rhizoma Musae*, Spearman correlation analysis was conducted on soil physicochemical properties, the top 50 differential bacterial genera, and the top 50 DEMs. A three‐tier association network was constructed using the selection criteria of |rho| > 0.7 and a *p*‐value threshold of < 0.01 (Figure 7), with larger circles representing higher degree values. The first layer encompassed five soil physicochemical factors, the second layer contained 24 rhizosphere bacterial genera, and the third layer comprised 26 metabolites from *Rhizoma Musae*. Among the soil physicochemical factors, AK, AP, and SOM emerged as significant potential influencers. AK showed positive associations with the relative abundance of OM190, TRA3‐20, Terrimonas, and MND1, and was positively correlated with the accumulation of (2E,4E)‐5‐(3‐(Hexopyranosyloxy)‐8‐hydroxy‐1,5‐dimethyl‐6‐oxabicyclo[3.2.1]octan‐8‐yl)‐3‐methylpenta‐2,4‐dienoic acid. AP exhibited positive correlations with OM190, TRA3‐20, and Terrimonas, while showing negative correlations with IMCC26256 and L‐Phenylalanine accumulation. SOM negatively correlated with the concentrations of L‐Isoleucine, L‐Valine, L‐Proline, and L‐beta‐homothreonine. Regarding rhizosphere bacteria, AD3 was found to have highly significant positive correlations with eight metabolites, including L‐Tryptophan, trans‐3‐Indoleacrylic acid, and Methyl‐1‐aminocyclopropanecarboxylate, while demonstrating a significant negative correlation with AK. OM190 was positively correlated with L‐Malic acid and p‐Coumaraldehyde, and negatively correlated with L‐Phenylalanine and L‐Valine, while also positively correlating with AP and AK. TRA3‐20 exhibited highly significant positive correlations with (2E,4E)‐5‐(3‐(Hexopyranosyloxy)‐8‐hydroxy‐1,5‐dimethyl‐6‐oxabicyclo[3.2.1]octan‐8‐yl)‐3‐methylpenta‐2,4‐dienoic acid and p‐Coumaraldehyde, highly significant negative correlations with L‐Phenylalanine and Methyl‐1‐aminocyclopropanecarboxylate, and significant positive correlations with AP and AK. In terms of metabolites, Methyl‐1‐aminocyclopropanecarboxylate was positively correlated with seven rhizosphere bacterial genera, including AD3, JG30‐KF‐AS9, and Subgroup_2, but negatively correlated with TRA3‐20, RB41, and Vicinamibacteraceae. These findings indicate significant associations among the metabolites of *Rhizoma Musae*, soil conditions, and rhizosphere bacteria.

## 4 Discussion

*Rhizoma Musae*, as a traditional medicinal and edible plant, has great potential in functional food development and nutritional research. The quality of such plant‐derived materials is closely related to their origin, and clarifying the association mechanisms of environmental factors on their quality is of great significance for guiding standardized production and improving utilization value. The origin of medicinal plants serves a pivotal function in determining their quality, with considerable variations in the content of medicinal compounds observed among herbs from diverse origins (Su, Wang, et al. 2023). Optimal climate conditions, soil properties, and microbial environments are believed to facilitate the synthesis and accumulation of bioactive compounds in medicinal plants, thereby improving their overall quality. In the present study, 16S rRNA high‐throughput sequencing and LC–MS methodologies were utilized to explore the impact of soil characteristics and rhizosphere bacterial communities from various origins on the quality of *Rhizoma Musae*.

### 4.1 Soil Factors Serve a Crucial Function in the Quality Formation Process of *Rhizoma Musae*

According to traditional Chinese medicine theory, medicinal materials sourced from specific regions are believed to exhibit superior quality and therapeutic efficacy, a concept reflected in the classification of such materials as “*daodi* medicinal materials” (Zhang et al. 2022). For example, patchoulol and pogostone, the principal medicinal constituents of *Pogostemon cablin* (Blanco) Benth, display variations in both total volatile oil content and genotype when cultivated in different cities within Guangdong Province (Zhang 2007). Soil factors have been recognized as key determinants influencing the quality of medicinal materials across various production areas (Xue et al. 2022). Research has shown that specific soil compositions exert a significant impact on the medicinal components of herbs. For instance, the presence of available soil iron (Fe) promotes flavonoid accumulation, while the levels of available manganese (Mn), total potassium (K), and AK are associated with the inhibition of flavonoid content in *Spatholobus suberectus* Dunn (Li et al. 2017). Previous studies have indicated that *Rhizoma Musae*, which is rich in flavonoids and alkaloids, contains active components with potential efficacy in the treatment of osteoarthritis (Zhang et al. 2025). Flavonoid compounds, being carbon‐based secondary metabolites, benefit from the accumulation of soil organic carbon and microbial biomass carbon (Rodríguez‐Arce and Saldías 2021). Alkaloids, as distinct bioactive compounds in medicinal plants, are known to exhibit anti‐inflammatory, cytotoxic, anti‐tumor, cytoprotective, gluconeogenic regulatory, and neuroprotective effects (Nie et al. 2016). Aibibaihan et al. (2021) reported that total flavonoid content varies across different species and regions, which is attributed to environmental factors and physiological distribution. The present study further corroborates these findings, revealing significant variations in the medicinal components of *Rhizoma Musae* from different origins, with *Rhizoma Musae* from GZ demonstrating higher flavonoid and alkaloid contents compared to other regions. Additional correlation analyses show that flavonoid accumulation in *Rhizoma Musae* negatively correlates with soil AP and AK content, while alkaloid content is positively correlated with soil pH. These observations indicate that soil physicochemical properties may be closely associated with the accumulation of medicinal components in *Rhizoma Musae*. We speculate that soil factors including pH, AP, AK, SOM, and AN showed significant associations with the composition and structure of rhizosphere bacterial communities, which had putative links with the biosynthesis and accumulation of secondary metabolites. This hypothesis needs strict experimental verification.

### 4.2 Bacterial Community Structure and Diversity in the Rhizosphere Soil of *Rhizoma Musae* Remained Relatively Stable

Overall, the bacterial composition and diversity in the rhizosphere soil of *Rhizoma Musae* from various origins were found to remain relatively consistent. At the phylum level, *Proteobacteria* exhibited the highest relative abundance in the rhizosphere soil of *Rhizoma Musae*. *Proteobacteria* are capable of synthesizing biotin precursors through the metabolic degradation of lysine (Hu and Cronan 2020). Biotin, an essential compound for all organisms, serves a vital function in the biosynthesis of fatty acids during carbohydrate metabolism, as well as in amino acid metabolism and carboxylation processes. Furthermore, *Proteobacteria* are known to produce a range of glycoside hydrolases, which facilitate the breakdown and utilization of organic matter, thus supporting nitrogen fixation (Su, Ji, et al. 2023).

The rhizosphere soils of *Rhizoma Musae* from five distinct origins were predominantly weakly acidic, with pH values ranging from 5.43 to 5.92. A dependency of most bacterial communities on specific pH ranges was observed, suggesting a strong relationship between soil pH and bacterial populations (Sun et al. 2015). For example, a negative correlation was identified between the relative abundance of *Proteobacteria* and soil pH (Yang et al. 2020), while *Acidobacteria* exhibited a positive correlation with pH levels (Wang et al. 2018). Phosphorus and potassium are regarded as critical nutrients for plant growth, promoting root development and serving as key regulatory elements influencing interactions between plants and soil microorganisms (Padalia et al. 2022). These nutrients have the capacity to modify both the microbial biomass and community structure within the soil (Li et al. 2023). Furthermore, it is hypothesized that various soil factors may influence the relative abundance of rhizosphere bacteria in *Rhizoma Musae*. At the genus level, rhizosphere soils from different origins of *Rhizoma Musae* were found to harbor distinct bacterial genera. For instance, the GX group exhibited seven differential genera, including *Acetobacteraceae*, *Pedosphaeraceae*, and *Edaphobacter*. Further correlation analysis revealed that AP content was positively correlated with the growth of *OM190*, *TRA3‐20*, and *Terrimonas*, while AK content showed positive correlations with the growth of *OM190*, *TRA3‐20*, *Terrimonas*, and *MND1*. *OM190* exhibits diverse biosynthetic potential for secondary metabolites, including antimicrobial compounds, and plays an ecological defensive role in its symbiosis with microalgae (Ludington et al. 2017). As key rhizospheric microorganisms in bioretention systems, *TRA3‐20*, *Terrimonas*, and *MND1* can improve rhizosphere nutrient status for vetiver and cattail by regulating nitrogen transformation, mobilizing organic phosphorus, and maintaining stable nutrient supply under hypoxic conditions, thereby promoting root development and plant growth while enhancing their adaptability in phytoremediation systems (Narayanasamydamodaran et al. 2025). These rhizosphere bacteria showed significant correlations with soil nutrient bioavailability and had putative associations with the precursor supply for secondary metabolite synthesis. Key differential genera including *AD3*, *OM190*, and *Subgroup_2* were significantly correlated with metabolites involved in phenylpropanoid metabolism and flavonoid biosynthesis pathways. We propose a hypothesis that these microbes are correlated with the metabolic flux and key catalytic steps of these pathways. Such microbial correlations may be associated with the accumulation of flavonoids, phenolics, and alkaloids, which showed significant differences in total flavonoids, polysaccharides, total phenols, and alkaloids among samples from different origins. This hypothesis remains to be verified by further experiments.

### 4.3 Medicinal Efficacy of *Rhizoma Musae* From Different Production Regions May Exhibit Variations

Among the four comparison groups, with the GX group serving as a reference, DEMs exhibiting substantial changes in content were identified. The physiological roles and physicochemical properties of these substances are likely to influence their medicinal efficacy, warranting further investigation. 4‐Hydroxy‐3‐(4‐hydroxyphenoxy)benzoic acid, a derivative of benzoic acid, was found to exhibit elevated levels in all four comparison groups relative to the GX origin. Although comprehensive pharmacological effects remain unreported, its molecular framework includes the benzoic acid backbone, with partial structural similarities to gallic acid (Deng et al. 2022) and protocatechuic acid (Tan et al. 2023), which may suggest possible pharmacological activities, including antioxidant and anti‐inflammatory properties. (1r,13s,15s,18s)‐5,7‐Dioxa‐12‐azapentacyclo[10.5.2.0^1^,^13^.0^2^,^10^.0^4^,^8^]nonadeca‐2,4(8),9,16‐tetraene‐11,15,18‐triol (also known as Yemenine C), a crinine‐type alkaloid, has been shown to markedly inhibit nitric oxide production in lipopolysaccharide‐activated macrophages (Abdel‐Halim et al. 2004). Since nitric oxide is known to serve a pivotal function in inflammatory processes, and its overproduction is associated with inflammatory responses (Moilanen and Vapaatalo 1995), this suggests the compound's potential anti‐inflammatory activity. The compound 7‐[(2S,3R,4S,5S,6R)‐4,5‐dihydroxy‐6‐(hydroxymethyl)‐3‐[(2S,3R,4R,5R,6S)‐3,4,5‐trihydroxy‐6‐methyloxan‐2‐yl]oxyoxan‐2‐yl]oxy‐5‐hydroxy‐2‐(4‐hydroxyphenyl)‐3‐[(2S,3R,4S,5S,6R)‐3,4,5‐trihydroxy‐6‐[[(2S,3R,4R,5R,6S)‐3,4,5‐trihydroxy‐6‐methyloxan‐2‐yl]oxymethyl]oxan‐2‐yl]oxychromen‐4‐one, a complex flavonoid, contains multiple cyclic structures and hydroxyl functional groups, with its chromene substructure resembling that of coumarin (Gupta et al. 2024). The integrated analysis of microbiome and metabolome further clarified the relationships between rhizosphere bacterial communities and specific metabolites in *Rhizoma Musae*. Based on Spearman correlation analysis, a three‐tier association network was constructed among soil physicochemical properties, the top 50 differential bacterial genera, and the top 50 differential metabolites. The results revealed that AK, AP, and SOM were the key influencing factors, which were significantly correlated with dominant bacterial genera such as *OM190*, *TRA*3‐20, *Terrimonas*, and *MND1*. These bacterial genera further showed significant correlations with the accumulation of specific metabolites, including p‐Coumaraldehyde, L‐phenylalanine, L‐tryptophan, L‐isoleucine, and L‐valine, which are closely involved in phenylpropanoid metabolism, flavonoid biosynthesis, and amino acid metabolism. Therefore, rhizosphere bacterial communities showed significant responses to soil nutrient changes and had significant correlations with the accumulation of specific secondary metabolites associated with the medicinal quality of *Rhizoma Musae*. These intricate structural features are likely to endow the compound with unique biological activities and physicochemical properties. It should be noted that the functional predictions of these substances must be confirmed through further pharmacological and clinical studies.

### 4.4 Limitations

This study has several limitations that should be acknowledged. First, this was an observational field study based on natural habitats of *Rhizoma Musae* from different geographical origins; thus, site‐specific environmental conditions and field management practices could act as potential confounding factors that were not fully controlled. Second, metabolite identification was performed using high‐throughput untargeted metabolomics, and structural validation of key differential metabolites was not fully completed with authentic standards. Third, the relationships among soil properties, rhizosphere bacteria, and plant metabolites were revealed only by association and correlation analyses, which can identify significant co‐variation but not confirm direct causal or regulatory mechanisms. Causal links and regulatory pathways were not experimentally verified by microbial inoculation, synthetic community tests, or gene regulation assays. Further studies with standardized cultivation, targeted metabolite quantification, and functional validation are warranted to strengthen the mechanistic understanding of quality formation in *Rhizoma Musae*.

## 5 Conclusions

This study investigated variations in the medicinal components of *Rhizoma Musae* from different origins, and elucidated the associations between soil factors, rhizosphere bacterial communities, and *Rhizoma Musae* metabolites. Although bacterial community composition and diversity were relatively stable across different origins, soil factors were significantly associated with the relative abundance of dominant bacterial genera and the metabolite profiles of *Rhizoma Musae*. Metabolomic analysis and pathway enrichment results revealed that differential metabolites of *Rhizoma Musae* from different origins were significantly enriched in key pathways, including phenylpropanoid metabolism, flavonoid biosynthesis, and ABC transporters. Specifically, *Rhizoma Musae* from different origins exhibited differences in the metabolic fluxes of upstream precursors for flavonoid synthesis and downstream functional flavonoid glycosides. This coordinated variation directly resulted in significant differences in the total content and component composition of flavonoid‐derived medicinal active ingredients in *Rhizoma Musae* across different origins. By constructing an association network encompassing “soil factors–rhizosphere bacteria–*Rhizoma Musae* metabolites”, we found that soil properties and rhizosphere microorganisms showed significant associations with soil nutrient uptake and utilization, as well as the regulation of plant signaling molecules. These factors were correlated with the metabolic pathways of *Rhizoma Musae* and its quality formation.

## Author Contributions

**Xinying Xiong:** methodology. **Jing Lei:** data curation. **Zhongze Wang:** formal analysis, supervision. **Xiaoyu Yang:** data curation. **Yae Yang:** formal analysis. **Fanzhi Liu:** conceptualization, resources, supervision, funding acquisition. **Xiaorong Zhao:** formal analysis. **Peiling Long:** data curation. **Wanyan Shen:** conceptualization, resources, funding acquisition, supervision. **Jiandong Liu:** data curation. **Jian Zhang:** conceptualization, methodology, writing – original draft. **Xuegang Luo:** methodology, writing – review and editing. **Yu Zhang:** conceptualization, resources, writing – review and editing, funding acquisition.

## Funding

Guizhou provincial program on commercialization of scientific and technological achievements ([2024]YB091). General fund of Guizhou Academy of Agricultural Sciences ([2024]10). China Agriculture Research System of MOF and MARA (CARS‐31). Guizhou Academy of Agricultural Sciences, Research on Innovation and Efficient Key Technologies of Characteristic Crop Germplasm in Guizhou Hot Zone (Qiannongke Germplasm Resources [2024] No. 08).

## Ethics Statement

The authors have nothing to report.

## Conflicts of Interest

The authors declare no conflicts of interest.

## Supporting information


**Table S1:** PERMANOVA results of overall differences in rhizosphere bacterial community structure of *Rhizoma Musae* among different origins.

**Table S2:** Metabolite information of *Rhizoma Musae* from five different origins.

**Table S3:** Differential metabolite information of *Rhizoma Musae* from five different origins.

## Data Availability

The raw sequencing data have been deposited in the NCBI Sequence Read Archive (SRA) under the BioProject accession number PRJNA1362626.
